# The psychedelic-peptide paradox: a hormetic hypothesis

**DOI:** 10.1016/j.cpnec.2025.100303

**Published:** 2025-06-02

**Authors:** C. Sue Carter

**Affiliations:** Kinsey Institute, Indiana University and Department of Psychology, University of Virginia, USA

**Keywords:** Psychedelics, Oxytocin, Vasopressin, Stress, Hormesis

## Abstract

The purpose of this narrative review is to examine the hypothesis that two neuropeptides, vasopressin (VP) and oxytocin (OT) and their receptors have central roles in the behavioral and physiological consequences of psychedelic interventions. Transient consequences of psychedelics can include anxiety and in some cases sickness responses such as nausea and vomiting, which may involve VP and other components of the hypothalamic-pituitary-adrenal axis. Stressful experiences are often followed by a pulsatile release of OT. The effects of OT depend on interactions with VP and may be more apparent following stressful experiences including those associated with psychedelic drugs. Effects of both the VP-OT system and psychedelics also are mediated through interactions with the autonomic nervous system and the immune system, contributing to a process called “stress response hormesis.” The hypotheses arising from a hormetic perspective could guide novel approaches to understanding dose- and time-dependent psychedelic functions and to the treatment of emotional and physical disorders.

## Highlights

• Both psychedelics and peptides influence reactions to trauma and chronic stress.
• Vasopressin and oxytocin function as a dynamic system affecting responses to stressors.
• Initial responses to many psychedelics produce transient “sickness” reactions involving vasopressin.
• Stressful experiences can be followed by a pulsatile release of oxytocin.
• Interactions of oxytocin and vasopressin also may help to explain the benefits and risks of psychedelics and hormesis.

## 1 An overview and hypotheses

Stress-related, chronic disorders, including depression and anxiety, have risen in the twenty-first century [1]. However, efforts to use knowledge of classical stress hormones or their receptors to treat anxiety or depression have not created effective medications for these disorders [[2], [3], [4]], and attention has recently turned to drugs collectively called “psychedelics.” (For details on the use of this term see Section 4.)

Despite enthusiasm for the clinical value of psychedelics their mechanisms of action are controversial and appear to be paradoxical [[5], [6], [7], [8], [9], [10]]. For example, the effects of many types of psychedelics include anxiety and nausea, described as “akin to an acute stress response,” followed by increased sociality, and in some cases euphoria and a sense of wellness [11]. This pattern of change is not fully explained by current theories of psychedelic action.

**Fig. 1:**
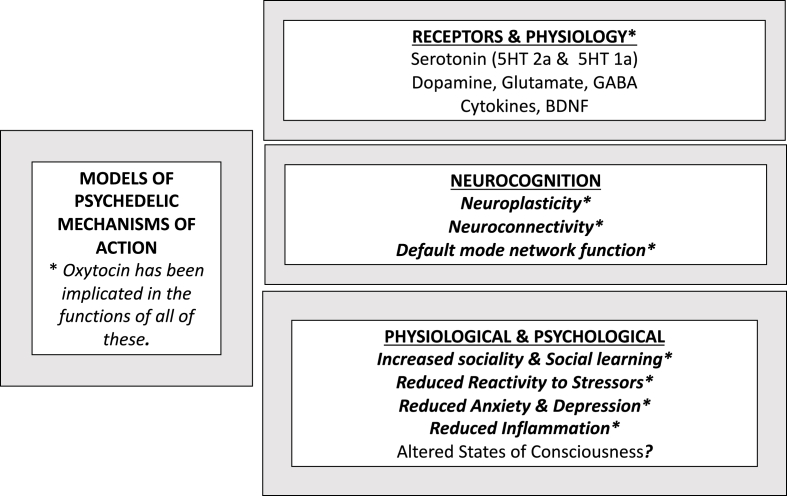
**Models of psychedelic mechanisms of action**. As detailed below, oxytocin has effects similar to those marked with ∗. Adapted from van Elk and Yaden [13]. See text below for details.

The neuropeptide, oxytocin (OT) and psychedelics can have a similar profile of physiological and behavioral consequences [12] (Fig. 1). OT levels also can be increased by psychedelics. Various psychedelics and OT have complex interactions with serotonin that may help to explain their interactions. (See Section 3.1). However, additional research is needed to distinguish possible differences in the effects on the VP-OT system of classical psychedelics, which may rely heavily on serotonin, versus atypical psychedelics, which have different mechanisms of action and may be less likely to induce emesis (See section 4.2)?

Among the shared effects of psychedelics and OT are reductions in reactivity to stressors [14,15], increases in sociality [12,16,17] and neuroplasticity [17,18]. Managing chronic inflammation and pain are other benefits proposed for OT [19], as well as psychedelics [[20], [21], [22]].

OT is a component of a neuroendocrine system that includes a similar molecule, vasopressin (VP). At present less is known about the effects on VP of serotonin [23] or psychedelics [24]. However, in contrast to OT, VP's effects generally have been associated with anxiety [25] as well as nausea and emesis [26]. Functions associated with VP parallel those reported after psychedelic use [11,[27], [28], [29], [30]].

The concept of stress has many definitions, but is broadly used to describe challenging events and processes with the potential to disrupt physiological or behavioral homeostasis. Research on stress originally focused on the HPA axis, including VP, corticotropin-releasing hormones (CRH), catecholamines and glucocorticoids [[31], [32], [33], [34]]. Autonomic, immune, microglial, mitochondrial systems and the microbiome are now recognized as critical components in adaptations to challenge [35,36].

Responses to psychedelics and interactions between VP and OT follow physiological patterns [11] that in other contexts have been called “stress response hormesis” [37,38]. Hormetic effects of psychedelics may involve sequential “stressful” effects of VP, followed by a pulsatile release of OT. This hypothesis is speculative and intended to encourage future basic research. However, beyond effects on social behavior [[39], [40], [41]], the possible roles of VP and OT in psychedelic functions have received little attention. The notion that hormesis involves the VP-OT system also is novel: a PubMed search conducted in April of 2025 crossing “hormesis” with either “oxytocin” or “vasopressin,” “retrieved zero results.”

## 2 Background on vasopressin (VP) and oxytocin (OT)

Both VP and OT consist of a six amino acid ring (held together by a disulfide bond), with a three amino acid tail. Among the major neural sources of OT and VP are the paraventricular (PVN) and supraoptic nuclei (SON) of the hypothalamus [42]. In the hypothalamus OT and VP are usually found in different cells, with different patterns of neurophysiological activity [43]. VP also is expressed in the suprachiasmatic nucleus and plays a role in circadian rhythms.

The functions of VP differ across time, following a temporal pattern similar to those described as acute versus chronic “stress.”

The effects of OT are more consistent, generally serving to buffer physical and emotional reactivity and promote healing and restoration.

VP and OT arose at different epochs in evolutionary history and have many diverse and interactive functions [19,[44], [45], [46]]. The genes regulating expression of VP and OT peptides are located adjacent to each other on the same chromosome (human chromosome 20) in opposite transcriptional orientation. Gene expression for VP and OT and their receptors is dynamic and the interactive characteristics of these change across the lifespan and according to environmental and social demands [[47], [48], [49]]. The consequences of OT typically involve down-regulation of the hypothalamic-pituitary-adrenal (HPA) axis [50,51], while, overtime, exposure to VP is associated with chronic stress [52] (Table 1).

**Table 1:** Functions of vasopressin and oxytocin: Time matters

FUNCTIONS or PROCESSES AFFECTED	VASOPRESSIN V1a Receptor	OXYTOCIN OT Receptor
**Supports prosociality – ACUTE**	**YES**	**YES**
**Supports prosociality – CHRONIC**	**NO**	**YES**
**Analgesic - ACUTE**	**YES**	**YES**
**Analgesic – CHRONIC**	**NO**	**YES**
**Anti-inflammatory - ACUTE**	**YES**	**YES**
**Anti-inflammatory – CHRONIC**	**NO**	**YES**
**Stress enhancing - ACUTE**	**YES**	**YES**
**Stress-buffering – CHRONIC**	**NO**	**YES**
**∗Dissociative states** **Increasing altered states**	**YES** **VP receptor (V1a?)**	**NO∗?** **OT receptor**

Legend: Categorical differences are presented for illustration and to open discussion. In vivo VP and OT serve as an integrated, adaptive system. However, the effects of VP and OT are receptor-, time- and dose-dependent. Acute, rapid effects are most commonly observed with VP V1a receptors, while the effects of OT and the OT receptor are slower and may be restorative. See text for additional references. ∗OT also may protect against vulnerability to altered states or intoxication [53].

### 2.1 Vasopressin and adaptation to challenge

VP is found throughout the body and is a central regulator of the HPA axis [2]. VP helps to connect the autonomic system with the demands of defense of self and others, including adjustments in behavioral homeostasis. VP also has a major role in anxiety [51]. VP has the capacity to augment the effects of CRH, potentially increasing reactivity to challenges [2,52]. For example, when functioning together CRH and VP amplify each other's effects [54]. VP also is synthesized in microglia, and may increase hypertension and reactivity to brain injury [55]. In this context, VP has been described as a “stress hormone.”

VP release is triggered by intense challenges, and potentially enhanced by a history of trauma or the absence of nurture, especially in early life [56,57]. Atypical functions in the VP-OT system have been implicated in various psychiatric conditions and disease states [24,58], including those associated with emotional dysfunction, psychotic symptoms and post traumatic stress disorder (PTSD) [59]. Other medical syndromes also are associated with VP secretion, including hyponatremia, and these can involve psychotic-like experiences.

### 2.2 Oxytocin has protective consequences

OT has many functions, going beyond effects on reproduction, to include mechanisms of protection and restoration throughout the entire body. Deficiencies in OT have been identified in many endocrine disorders [60]. Exposure to OT may have a particularly relevant role in encouraging social interactions and attachments [61], as well as the resolution of reactions following negative social experiences [62]. The pulsatile release of OT following intense experiences can be protective, restorative and capable of resetting its own system through positive feedback.

OT may help to moderate reactivity to CRH and VP, with consequences for reducing physiological reactivity and projecting accurate responses to future threats [63]. OT has many levels of interaction with the HPA axis, including adrenal glucocorticoids, generally inhibiting over reactivity in that system [25].

OT has a documented role in neural development [64] and neuronal plasticity across the lifespan [65,66]. OT also has a role in stem cell differentiation, and is capable of programming cell fates, including apoptosis [67]. For example, in the hippocampus OT increases neural plasticity, while VP apparently does not [68]. Microglia also are regulated by OT; with a role in neural plasticity [69,70]. OT also can be found in microglia [71], where it is released by stressful experiences.

OT supports cortical connectivity [72]. Furthermore, OT has been implicated in tissue maintenance and repair [73]. Exogenous OT can reduce electrical activity in the default mode netword (DMN). OT effects on the DMN are seen even in rodents, and OT can increase connectivity among cortical areas [74]. Neuronal connectivity is affected by OT in ways that interact with, but differ from VP [72,75,76].

OT supports synchrony, both within bodily systems [12,77] and among individuals [62]. Pulsatile release of OT is of particular importance in eliciting rhythmic contractions of visceral muscles, including those necessary for milk ejection, childbirth, ejaculation and orgasm [12,78]. Chronic VP may have asynchronous consequences including facilitating premature labor [79].

Research in rats suggests that the OT receptor can be upregulated by stressful experiences or corticosterone treatments [80]. In contrast, tissue damage after injury may involve **chronic** exposure to VP [52], activation of the VP receptor, or other lasting changes that could lead to oxidative stress and disruptions in function. These in turn are potentially prevented or even reversed by OT [19]. Based on this hypothesis, psychedelics capable of increasing endogenous OT or its activity at the OT receptor, and/or reducing exposure to VP could reduce the effects of chronic stress or protect from other forms of brain disruption, including hypoxia [81].

Many of the benefits attributed to psychedelics described below, including (3,4-methylenedioxymethamphetamine] MDMA, are similar to reported effects of OT (Fig. 1). However, high doses of OT, such as those released after MDMA [82], also can bind to the V1a receptor. Slower, and potentially more lasting positive effects of OT, may rely on the OT peptide or stimulation of the OT receptor. This hypothesis has not been well studied, but has many implications.

### 2.3 Oxytocin-vasopressin receptor interactions

OT is traditionally described as having one “primary” receptor gene, the *oxtr* [83,84]. Three separate G-protein coupled receptors (GPCRs) also have been identified as primarily VP receptors. Of these the gene regulating expression of the VP 1a receptor has been most studied in the context of behavior, stress and depression [85]. The VP 1a receptor has a high affinity for VP, but also can bind OT [86,87], with effects that may vary across the sexes, among individuals, across the lifespan and among brain regions [88,89].

Cross-talk occurs among the OT and VP peptides and their primary receptors [61,85,90]. Receptors for VP and OT have been described throughout the nervous system [58,91], as well as the digestive system [92], kidney [93], the immune system [94] and on astroglia [71]. OT and VP activate GPCRs with different subcellular signaling processes and time courses [95]. Furthermore, both OT and VP can affect ion receptors, presumably with different consequences [96].

Both OT and VP receptors are co-expressed with and function in conjunction with CRH [63]. A second vasopressin receptor, the VP 1b receptor also has been implicated in managing reactions to challenges in both the pituitary, brain and kidney [97,98]. The VP 1b receptor also interacts with serotonin, and is a candidate for pharmacological stress-management [4].

VP and OT both act on the autonomic nervous system as part of a brainstem system, also involving serotonin, that allows volatile rejection of poisons and conditioned aversions. The VP V1a receptor, as well as the OT receptor and serotonin can have direct effects on visceral contractions, including those leading to vomiting (M. S. [99]).

There are many other potential opportunities for OT and VP to interact with each other, with consequences that are only now being recognized. For example, a recent study in rats revealed that the effects of OT and VP in the bed nucleus of the stria terminalis involved their shared capacity to stimulate a specific type of cell (described as a TYPE III cell). Type III cells contain both OT receptors and receptors for CRH [88].

Because of its capacity to form complexes with many other types receptors, the OT receptor has been described as the “hub” for transmembrane GPCRs [100,101]. Heteroreceptor complexes allow different types of molecules to work together and to adjust subcellular signaling and physiological functions, with both excitatory and inhibitory capacities. Effects on subcellular signaling also may help to explain shared adaptive functions of the VP-OT system as well as interactions with components of the HPA axis [95] and, as argued here, the cellular consequences of psychedelics.

## 3 Serotonin

### 3.1 Serotonin, psychedelics and peptides

Serotonin and its receptors, especially the 5HT-2a and 5HT-1a receptors, are the best studied targets for psychedelics and have dominated the study of psychedelic function [10,18,40,102]. These theories were originally based on the hypothesis that serotonin is released by psychedelics with behavioral effects focused on 5HT 2a and 5HT 1a receptors. The effects of psychedelics extend to other serotonin receptors, most of which are not well studied [103]. However, stimulation or blocking of specific serotonin receptors alone is not sufficient to explain the complex effects of psychedelics and many other neural systems presumably play a role in the consequences of psychedelics [6].

Serotonin is an indoleamine, synthesized from the essential amino acid, tryptophan [104]. The majority of serotonin synthesis occurs in the digestive system and in red blood cells, while less that 10 percent of serotonin is created in the brain. Serotonin *of neural origin* is synthesized primarily in the brainstem (raphe nucleus) with projections to both the neocortex and hypothalamus [42]. Serotonin availability also is managed in part by selective serotonin transporters (SERTs) [105]. SERTs influence the duration of a serotoninergic effect and allow serotonin to be recycled. SERTs are also potential targets for psychedelics.

The effects of serotonin are receptor-dependent, brain-region specific and dynamic [106]. Serotonin receptors form heteromers with the OT and possibly VP receptors, as well as with receptors for dopamine, opioids and other psychotropic molecules [101,107].

In the nervous system, serotonin usually functions in synchrony with OT [[108], [109], [110]]. OT has been shown to release serotonin and serotonin can release OT [111]. OT also can increase the availability of serotonin receptors and may increase heteromers of serotonin receptors with the OTR [101]. Drugs capable of directly increasing serotonin, such as fenfluramine, also have been shown to increase neural activity in hypothalamic OT producing neurons [112].

OT-serotonin interactions support sociality [40,109,110], modulate reactivity to stressors [113], and can influence digestion [108]. However, over time, serotonin may inhibit OT as well as the effects of OT, such as the capacity for orgasm [114]. Interactions with byproducts of serotonin metabolism, including kynurenine pathways, may influence both positive and negative effects of OT [113].

Serotonin also can release VP, but VP-serotonin interactions are receptor dependent and vary among the various 5 HT receptor subtypes [103]. In conjunction with other HPA axis hormones, VP also is capable of reducing the central release of serotonin [54]. Serotonin also may inhibit some functional effects of VP ([115]; Ferris, 2008), possibly indirectly supporting the effects of OT. When initially taken SSRIs are associated with an increase in OT [50]. However, the chronic effects of SSRIs may involve downregulation of serotonin receptors and reduced effects of serotonin [114].

### 3.2 Serotonin-based medications can affect psychedelic activity

Interactions of the serotonin system with the OT system may help to explain some effects of psychedelics. For example, medications that affect the serotonin system also could alter the clinical impact of psychedelics.

Many individuals treated with psychedelics are receiving SSRIs or have a history of SSRI use. SSRI discontinuation and withdrawal symptoms makes the interpretation of serotonin-psychedelic interactions more complex [116]; this is especially problematic if these drugs are stopped abruptly [[117], [118], [119], [120]]. For example, an analysis of the effectiveness of MDMA in the treatment of PTSD indicated that “*recent use of SSRIs dampened the effectiveness of MDMA”* [121]. It also has been reported that the anti-depressant benefits of psilocybin were “*reduced in individuals who had discontinued SSRI use immediately prior to psilocybin”* treatment [116].

The consequences of both psychedelics and serotonin affect essentially every bodily system [122]. Moreover, a variety of other neurochemicals including dopamine, opioids, GABA/glutamate, acetylcholine, BDNF (brain-derived neurotropic factor) as well as cytokines and other components of the immune system also contribute to the effects of serotonin and have interactions with OT, VP and psychedelics [12,40] (Fig. 1).

Dysfunction in the serotonin system, including deficiencies, excess or withdrawal from serotonin-based medicines, can have negative behavioral consequences including increases in anxiety and depression and disruption of digestion. For example, nausea has been attributed to the consequences of increasing serotonin (acting on 5HT-3 receptors) and a slowing of digestive functions [123,124]. There are indications that serotonin contributes to nausea and vomiting, possibly through effects on the area postrema [26]; these effects would also involve the vagus. VP-OT-serotonin interactions could contribute to the “come-up” phase of psychedelic use, described below.

There are many points of interaction among serotonin and the VP-OT system. Although beyond the scope of this review, it is useful to keep in mind that the VP-OT system as well as serotonin pathways vary genetically and are epigenetically calibrated by a history of adversity or nurture [125,126]. The interactive effects of OT and VP may diverge depending on these factors.

## 4 Background on psychedelics

### 4.1 What exactly is a psychedelic?

Compounds with the capacity to alter human consciousness and influence emotional states have been available for thousands of years. These molecules, sometimes loosely combined under the term “psychedelics,” were components of healing and spiritual traditions. In the 20th century psychedelics came into wider use as recreational drugs [127].

In common usage the term “psychedelics” describes a variety of compounds functionally capable of influencing neural function and, in some cases, inducing altered states of consciousness. Some of the contemporary molecules used to induce psychedelic experiences are naturally occurring compounds and are based on traditional medicines. Traditional psychedelics are mixtures of molecules with effects on multiple tissues through the body [128]. This especially applies to products extracted from plants (such as fungi or cacti), but psychedelically-active substances also are derived from some animals (such as toads) and others are synthetic in origin.

Several drugs with psychedelic properties have been synthesized in laboratories over the last century [10,127]. However, it is important to note that **both traditional and synthetic psychedelics act through effects on endogenous systems**. These endogenous molecules, including neuropeptides and neurotransmitters, in turn have characteristics of their own that may help to explain the effects of psychedelics.

The most commonly discussed targets for psychedelics are in the central nervous system. However, as with the VP-OT system, psychedelic drugs also have documented functions on the autonomic nervous system [14], immune system [22], kidneys, digestive system, glia, mitochondria and microbiota.

### 4.2 Classical and atypical psychedelics

Among molecules classified as “classical” psychedelics are lysergic acid diethylamide (LSD), N,N- dimethyltryptamine (DMT) [129], psilocybin, and mescaline [130,[130], [131], [132],^132^]. Other drugs with psychedelic-like properties were originally synthesized for non-neural applications including prevention of hemorrhage [e.g. MDMA [13,133,134].

Psychedelics that release and/or act on serotonin receptors have been called “serotonergic psychedelics.” Ibogaine [135], 5-MeO-DMT [136] and MDMA [127] are sometimes called “atypical” psychedelics, with functional effects that are different from the classical “serotonergic” psychedelics. For example, MDMA seems to be less likely than serotonergic psychedelics to trigger mystical experiences and nausea, and more likely to increase sociality and reduce reactivity to negative experiences [137,138]. In this context, a polypharmic approach, sometimes called “hippy flipping” or “candy flipping,” arose from recreational drug use and appears to be widely practiced. This involves mixing psychedelics such as psilocybin or LSD plus MDMA, with the intent of moderating negative effects including “fear, grief and anxiety” [139]. For example, MDMA in comparatively low doses, perhaps by upregulating OT [41,140], appears to reduce adverse effects generated by serotonergic psychedelics [138,139]. Interactions of serotonin in conjunction with VP and OT, are not well studied, but may be important to the functional understanding of many psychedelics, including ketamine [141].

### 4.3 Ketamine

Ketamine, termed an “atypical psychedelic,” currently is medically used for the treatment of depression and anxiety [142]. As discussed here ketamine has several functional parallels with OT and classical psychedelics and MDMA, but also differences in mechanisms of action [141,143].

Ketamine, was initially developed as an anesthetic and at high doses can induce dissociative states and hallucinations [144]. Ketamine is an NMDA antagonist with consequences in the glutamate-GABA system. Ketamine also can increase BDNF. At subanesthetic doses ketamine increases activity in the serotonin system, possibly by inhibiting the serotonin transporter [145] or by increasing expression of serotonin receptors [141,146]. Ketamine also may act through serotonergic-kynurenic pathways, where it reduces inflammation [22].

Ketamine increases sociality in both humans and rats, possibly by lowering anxiety and altering threat responses [147]. However, ketamine has potential for abuse, possibly in part due to gradual down-regulation of endogenous OT pathways. In animal models the behavioral effects of long-term ketamine use were reversed by OT treatment [148]. Excess or prolonged use of ketamine also may cause kidney damage [149], indirectly suggesting a possible connection to VP or its receptors. Several of the consequences of ketamine resemble known functions of OT [17]. However, in general, especially given its wide clinical use, the roles of both VP and OT in ketamine's function need additional study.

### 4.4 Do psychedelics release OT?

Significant increases in OT levels in blood have been measured following treatment with a variety of psychedelics-like drugs. For example, there is evidence of a release of OT following MDMA [39,82,150,151], LSD [152], DMT [129], 5-MeO-DMT [136], mescaline [153], psilocybin [154] and ketamine [141].

In the case of MDMA administration, the timing of a significant release of OT into blood seemed to coincide with the onset of effects on social behavior, with both initially occurring within 60–90 min [154]. Whether other psychedelics, such as psilocybin (with a faster behavioral onset than MDMA), have different temporal consequences for OT release needs additional study.

Research, done mostly in male rats, supports the capacity of MDMA to release OT and to stimulate hypothalamic OT neurons. However, MDMA may influence OT release via a subdiaphragmatic vagal pathway to the intestinal microbiome [151]. The role of the vagus in the functions of psychedelics, including MDMA, needs more study since this also may help to explain the effects of that drug on behavioral, digestive and immune functions.

### 4.5 Do psychedelics affect VP release?

MDMA can release VP [39,155,156]. In rats VP increased after MDMA treatment followed a pattern in blood that appeared similar to that seen in OT. However, following their initial secretion VP and OT have different temporal properties [157,158]. VP is difficult to measure and some studies in human have used copeptin as a surrogate for VP. However, copeptin is more stable than VP, complicating the use of copeptin as an index for *temporal changes* in VP following psychedelic use.

Based on the behavioral phenotypes of VP's effects and the side effects of many psychedelics it is plausible that psychedelic drugs have effects on VP and its receptors. However, the time-related and long-term effects of psychedelics on VP and OT need additional study since drugs with different properties have diverse effects on these peptides and their receptors. In addition, the effects of different psychedelics and dosages of drugs with different temporal profiles of action complicate the analysis of the functional effects of these molecules.

VP-serotonin interactions may be of particular importance to understanding both immediate and lasting risks or side effects of some psychedelics. For example, preclinical research suggests that under some circumstances these interactions are antagonistic, with VP preventing or reversing some of the effects of serotonin, as well as those of OT ([115]; [159]). As described above (Table 1), VP is associated with anxiety and lasting dissociative states. OT, presumably acting on the OT receptor, may be protective [160,161], possibly preventing detrimental effects of psychedelics and enhancing benefits.

### 4.6 Context and individual differences

The effects of psychedelics vary among individuals [7,162]. Actions on the VP-OT system also could help to explain differential outcomes reported when psychedelics are taken in a context of perceived safety versus threat. Psychological, and social factors, sometimes called “set and setting,” can alter the consequences of a given psychedelic experience [138]. The physiological basis of “set and setting,” may be better understood through awareness of the VP-OT system, with many connections to emotional context [163] and the autonomic nervous system [164].

## 5 Models for psychedelic functions

### 5.1 Bottom-up perspectives on psychedelic

Bottom-up interpretations of the effects of psychedelics have focused on brainstem, hypothalamus [13,14,165,166], and the autonomic nervous system [18]. The hypothalamus as a primary site for the production of peptides, including VP and OT, has functional connections to peripheral physiology in part through the posterior pituitary gland, as well as effects on the sympathoadrenal axis and autonomics [17]. OT of hypothalamic origins reaches various areas throughout the nervous system. Thus, hypothalamic systems can influence both neocortical areas and the brainstem with potential consequences for coordinating cognitive, emotional and autonomic functions [167]. Although VP and OT also can affect cortical functioning, in the context of psychedelics, these peptides have most typically been studied as components of bottom-up perspectives. Thus, peptide perspectives have more often been associated with emotion, homeostasis and reactions to stressors.

### 5.2 Top-down perspectives on psychedelic functions

The capacity of psychedelic drugs to induced altered states of consciousness initially focused attention toward neocortical structures and cognition (Fig. 1) [13,168]. The consequences of psychedelics often have been attributed to changes in “top-down” cognitive processing, sometimes presumed to be neocortical in origin.

Among the possible effects of psychedelic drugs are the capacity to reduce repetitive thoughts and obsessions, including effects on the DMN. According to this schema when the DMN is overactive intrusive thoughts emerge. In a recent review of existing literature, Gattuso and colleagues [166] conclude, “Across psychedelics there is consistent acute disruption in resting state connectivity within the DMN and increased functional connectivity between canonical resting-state networks.” This interpretation of the effects of psychedelics focuses on the hypothesis that disrupting the repetitive or obsessive functions attributed to the DMN allows restoration of cortical flexibility or connectivity [168]. Psychedelic effects on consciousness also have been associated with reduced neural activity in the DMN [169]. In some models the anterior cingulate and insula are included in the DMN and may be involved in processes described as either “top down” or “bottom up” [166,170].

Neural imaging studies report that drugs such as psilocybin or LSD cause desynchronization of functional connectivity in both the neocortex and subcortex. This includes altering connections between the hippocampus and DMN [171]. For example, disruption in the functions of the DMN associated with LSD use may reduce intrusive thoughts, but also might allow hallucinations and psychological dissociation to occur, including negative cognitive experiences [172,173].

### 5.3 Time matters - temporal effects of psychedelics: “come-up” and “come-down”

The trajectory of emotional and physiological responses to classical psychedelics tends to follow a temporal pattern [11]. “Come-up” (onset) of psychedelic effects involves “negatively-valanced experiences, including nausea, anxiety, restlessness, confusion and social withdrawal.” During the come-up, compounds such as those found in ayahuasca or psilocybin can induce vomiting or anxiety. The come-up symptoms, including emesis, can be distressing and may last for 1–2 h.

“Come-down” may be associated with “distress-resolution,” including pleasant sensations, calm and relaxation, philosophical thinking, and social empathy or gratitude. Come-down from psilocybin reportedly lasts 2–4 h or longer [11]. However, drug-induced changes and flashbacks, either positive and negative in valence, are sometimes experienced for weeks or months follow a psychedelic experience, suggesting neural consequences that outlast the acute drug effects [10,174]. Whether the long-term use of psychedelics is associated with persistent changes in either VP or OT or their receptors does not appear to have been studied. However, in the case of chronic ketamine use, the OT system may become down-regulated, leaving the ketamine user in “withdrawal” [148].

## 6 Cognition

### 6.1 Psychedelics and cognition

Cognition is inherently difficult to define and model, especially in nonhuman animals. Human research on this topic often is based on neural correlates of state changes and subjective reports, while psychedelic research in animals may rely on motor patterns, such as “head twitch” responses, often attributed to the effects of serotonin [175].

The psychological effects of psychedelics in humans also have been correlated with changes measured by imaging and electrical activity across the nervous system [168,176]. It has been suggested that some psychedelic treatments facilitate the transmission of neural information or coupling among brain areas, thus potentially supporting “mental flexibility or creativity” [176,177]. For example, studies of brain activity after psilocybin, measured by fMRI, revealed decreased activity in anterior cingulate and medial prefrontal cortex and decreased coupling between medial prefrontal cortex and posterior cingulate. These findings were interpreted as evidence of “decreased activity and connectivity in the brain's key connector hubs, enabling a state of unconstrained cognition” [18]. More crudely stated, under the acute influence of psychedelics the neocortex seems somewhat disconnected from the brainstem.

### 6.2 Altered states of consciousness

In some cases psychedelics induce altered states of consciousness, including dissociative states, hallucinations and mystical experiences [14,178]. Altered states of consciousness are not unique to psychedelics, and also have been reported in holotropic breathing and “near-death experiences” [179,180].

There also are phenomenological parallels between drug-induced hallucinations and the psychoses reported in schizophrenia. However, the underlying neurobiology of these may not be identical. For example, the experiences associated with schizophrenia include effects on the auditory system, while psychedelic hallucinations are reportedly more likely to be visual [181]. In schizophrenia, unchecked hallucinatory experiences are more common in individuals with previous psychotic episodes and/or a history of trauma and adversity. Under some circumstances, especially in vulnerable individuals, psychedelic use also has been associated with worsening of preexisting conditions or the emergence of novel, distressing psychological symptoms [7,131,182].

The physiological and psychological changes that constitute vulnerability to psychotic experiences and enduring negative reactions to psychedelics are open to debate. However, recent studies in nonhuman models suggest that some of the neural benefits of psychedelic-assisted therapies can occur without disruptions in consciousness [[183], [184], [185]].

Exogenous VP and OT by themselves apparently do not induce psychosis, although psychotic states may release both peptides. Importantly, in vulnerable individuals **endogenous OT may be associated with a reduced risk of psychotic breaks** [160,186]. VP has been associated with increases in psychosis. For example, in unmedicated patients diagnosed with schizophrenia, comparatively high levels of VP were measured in some female patients experiencing “positive” symptoms (including hallucinations and delusions) [187]. Patients diagnosed with psychosis and/or hyponatremia, possibly including low levels of OT, also might be exceptionally sensitive to the capacity of endogenous VP to induce dissociative states [188]. It is possible, especially in the face of a challenge, that endogenous OT provides protection against both psychosis and the negative effects of psychedelics, as well as the chronic or unchecked effects of VP [52,186].

## 7 Plasticity and neural reorganization

Specific psychedelic effects on the nervous system reportedly include neural growth, synaptic plasticity and structural reorganization (Fig. 1). Changes in serotonin and its receptors [189] have repeatedly been described as mechanisms through which psychedelics increase plasticity and alter previous consequences of trauma [184,190]. Molecules, often focused on the serotonin system and sometimes termed psychoplastogens, are being created with the intent to increase neural plasticity without producing altered states of consciousness.

Another form of plasticity involves communication across cells by a process described as intercellular communication or “metaplasticity.” In theory, disruptions of function by psychedelics allow reorganization and healing of neural systems that have become overactive [191]. This model posits that disruptions could be followed by increased neural plasticity [190] or reopening metaplastic processes including “windows for social reward” [12,40].

An important and somewhat unique feature of OT's action is it capacity to exhibit positive feedback and thus escalate its own pulsatile release [17,61]. This may help to explain how or why some psychedelics have long-term beneficial consequences. However, as described below, the benefits of psychedelics or OT may not be detected in the absence of a prior stressor, possibly due in part to interactions of VP and OT. The temporal interactions between OT and VP remain only partially understood and need further study.

## 8 Autonomic substrates of psychedelic functions involve neuropeptides

The autonomic nervous system, including the parasympathetic and sympathetic systems, are important to the execution and interpretations of both top-down and bottom-up perspectives on psychedelic functions [14]. For example, autonomic responses, including those associated with the release of **both** VP and OT, may be critical for the perceived “peak” experiences or alter states of consciousness described in certain kinds of psychedelic use. Both VP and OT are components of other kinds of euphoria experiences, including those associated with sexual behavior and forming new relationships [78,192].

Based on its' role in the HPA axis and sympathomimetic functions, we can speculate that VP's effects might be being more rapid than those of OT, with OT having slower and potentially enduring benefits [193]. This could contribute to the come-up component of psychedelic use. However, whether the effects of VP and OT are transient or long-lasting also depends on factors such as individual differences and a context of safety or threat [56].

The VP-OT system has a major role in the regulation of the parasympathetic nervous system, supporting various adaptive activities [17,50]. The autonomic nervous system also has the potential to directly and indirectly influence cognition, mood, emotional reactivity [164] and the immune system [118]. OT, VP, the parasympathetic nervous system and, in some cases, psychedelics can have effects through changes in inflammatory processes [22]. In another example, effects of OT can be mediated by the vagus nerve, which monitors changes in the intestinal microbiome [194]. Rodent models have implicated the vagus and/or the microbiome in the capacity of MDMA to release OT. Thus, cognitive and emotional effects of psychedelics, including MDMA, may be due in part to stimulation of vagal pathways [151].

## 9 Social effects of psychedelics

Psychedelic drugs capable of increasing social behavior have been called entactogens (intrapersonal), empathogens (interpersonal) or connectogens (“producing a joining together”) [195]. Some psychedelics, as well as MDMA, have particularly profound effects in reducing negative perceptions and increasing the rewarding value of social interactions [13,196], such as social touch.

The regulation of social behavior involves complex interactions between VP and OT [12,17,61]. OT and at least some psychedelics increase positive social behaviors [10,41,196]. VP also can affect sociality, but these effects are more transient and less predictable [90].

The capacity of MDMA, to increase sociality has repeatedly been associated with increased activity in the OT system [197,198]. Based on research in humans, it has been suggested that MDMA and related drugs may function to reduce reactivity to negative stimuli, rather than simply increasing prosocial behaviors [137]. For example, research in rodents suggested that the effects of OT, when released by MDMA, involved stimulation of the VP V1a receptor. OT can act as a VP V1 agonist or antagonist [82,150]. It is possible that OT, released by MDMA, could block VP receptors, thus preventing or reversing chronic or negative “side effects” of psychedelics, as well as reducing detrimental effects of chronic overactivation of the HPA axis [139].

## 10 The adaptive significance of nausea and vomiting

Anxiety, nausea and, in some cases, vomiting are reported during the initial exposure or “come-up” phase of psychedelic use [11]. These responses offer clues to the physiology of psychedelic actions. In an evolutionary context, an initial sickness response [30,36] would allow potentially dangerous substances to be expelled from the digestive tract.

The autonomic and behavioral experiences described in come-up are similar to functions attributed to excess VP [58] and/or a VP receptor system potentially sensitized by trauma [59]. VP can amplify the acute effects of stressful experiences including the release of other hormones of the HPA axis [2], while (over time) reducing the central release of serotonin [54].

Psychedelics, with the capacity to elicit a sickness response, defined by nausea and vomiting [155,156], can induce a physiological release of VP as well as increases in other components of the HPA axis and in components of the immune system. Nausea from various origins can release additional VP [199]. Functional changes in the HPA axis and/or serotonin also may contribute to the come-up component of psychedelic use. However, as described below, a hormetic response to psychedelics, including digestive distress, and associated increases in VP, might set the stage for a release of OT and/or behavioral and epigenetic effects described in the come-down experience. OT in turn could have lasting compensatory effects, supporting the come-down experience, but with beneficial consequences of its own (Fig. 1).

## 11 A hormetic hypothesis in the context of peptides and psychedelics

**“***Hormesis is a fundamental component of adaptability, neutralizing many endogenous and environmental challenges by toxic agents, thereby enhancing survival. Hormesis is highly conserved, broadly generalizable, and pleiotrophic, being independent of biological model, endpoint measured, inducing agent, level of biological organization and mechanism”* [200].

Hormetic effects are typically time- and dose-dependent, may be biphasic and can have sequential risks and beneficial consequences [201]. Here I suggest that awareness of the properties of hormesis could be of value in attempts to conceptualize the role of the VP-OT system in the phenomenology of psychedelic functions (Fig. 1). For example, time-dependent interactions among VP and OT could have critical roles in the temporal changes seen with psychedelic use [11].

### 11.1 What is stress response homesis?

*…” The principle of stress-response hormesis is nicely captured by the well-known maxim of the nineteenth-century German philosopher Friedrich Nietzsche: That which does not kill us makes us stronger.”* [201]*.* Based on the biology of psychedelics, sickness and other hormetic processes, perhaps Nietzsche should have said *That which make does not kill us, but does make us feel sick, may make us stronger?*

Hormetic responses are not restricted to psychedelics (Table 2). Increases in OT, indicated by either measurements of systemic OT or effects on the uterine contractions, also are associated with a variety of other potentially hormetic experiences including birth [79], sexual orgasm, love and the formation of attachments [78,193], vigorous exercise and social play, and psychosocial stress [202]. Compounds such as castor oil, which have emetic and also anxiolytic effects, also can facilitate human birth, presumably through the release of OT [203].

**Table 2:** Examples Of Stress Response Hormetic Processes which may involve vasopressin (VP) followed by pulses of oxytocin (OT)∗.

Vasopressin is associated with anxiety, nausea, vomiting and can be pro-inflammatory,	Oxytocin is associated with increased neuroplasticity, reductions in anxiety and is anti-inflammatory
Psychedelics – “Come-up” esp. DOI, LSD, psilocybin, ayahuasca, ibogaine	**Psychedelics – “Come-down” esp. MDMA, ketamine**
Early stages of parturition	**Postpartum euphoria and bonding**
Intense exercise, Vigorous social play, Thermal stress, Intermittent fasting	**Post-stress relaxation or euphoria**
Bulimia	**Post-bulimic anxiolysis**
GLP-1∗∗ receptor agonists (for example, as in *Ozempic*)	**GIP∗∗ may calm nausea: Is OT involved? (for example, as in *Mounjaro?*)**

Legend: ∗ Exposure to various drugs and experiences can elicit strong reactions, including anxiety, nausea and vomiting – all of which have been associated with VP [26]. Stressful experience are also routinely followed by an increase in OT [202], which may contribute to improvements in mood, emotion and sociality. Both VP and OT may be present in early stages of a hormetic response and may function in synchrony to allow a rapid adaptive response. The second stage of stress response hormesis would depend on the availability and buffering capacity of OT.

∗∗ GLP-1- glucagon like peptide −1: GIP - gastric inhibitory polypeptide.

### 11.2 Sources of individual differences in psychedelic and hormetic responses

Biological responses differ among individuals, and are sex, age, dose and context dependent. Many questions remain of possible relevance to hormetics and psychedelics. For example, sex differences are understudied in the analysis of psychedelic functions [191,204]. Sex differences and gonadal steroids also play a key role in mammalian hormesis, but are frequently overlooked or ignored [205]. During recreational use physiological side effects of psychedelics may be more pronounced in women. This could be due in part to sex differences in the capacity to manage excess VP, and/or differences in kidney function [206] and mechanisms underlying water balance [58].

### 11.3 Could hormesis and the immune system help to explain failed attempts to show effects of oxytocin or psychedelics?

The hypothesis that psychedelics are acting through a hormetic process brings up other questions that may be relevant to apparent failures of therapeutic attempts to use either psychedelics or exogenous OT. For example, in rodent models, stressful experiences uncovered benefits of OT that were not detected in the absence of a stressor [207]. It also has been reported that attempts to facilitate sociality with exogenous OT were most successful in the presence of challenges. For example, in rats OT significantly facilitated maternal behavior in novel, but not familiar environments [208], possibly due to interactions of OT and hormones of the HPA axis. In mice low doses of LSD were able to restore normal function in animals that had experienced a repeated stressor; this treatment did not have a measurable effect in unchallenged animals [152]. In humans, LSD (at a low doses) was shown to have more pronounced effects in individuals experiencing higher levels of depression [209].

It has been reported that individuals with a history of adversity or clinical disorders may be particularly likely to gain long-term benefits from psychedelics [10,162,210]. These observations could be due in part to the “law of initial values.” That is, there is more room for improvement when you start low. However, it is also possible that an intense reaction of the HPA axis, including the release of VP or the actions of glucocorticoids or other HPA axis hormones, could potentially facilitate a pulsatile release of OT and upregulate the OT receptor [80], with hormetic consequences?

Another possible connection between stress-related disorders, such as depression [36], and hormesis comes from the fact that components of the immune system are capable of influencing the effects of VP and OT and can be affected by psychedelics. The relationship between immune responses and the benefits of OT may occur in part because molecules such as the receptor for advanced glycation end-products (RAGE), immunoglobulins and the complement system help to transport peptides and can determine access to the brain through the blood-brain barrier [211]. The blood-brain barrier also may be more easily penetrated following stress. The sequential roles of VP and OT in managing inflammation could be of particular importance to understanding the time-dependent and biphasic effects of both peptides and psychedelics [22,118].

Time-related and persistent effects of psychedelics and peptides are especially difficult to study and have had less attention than acute changes (Table 1). The complex interactive functions of VP and OT could play a pivotal role in experiences described after psychedelic use. These would be expected to be time, experience and context dependent. For example, it is common for psychedelic experiences to be followed by a period of mild depression, resembling a “hangover” – possibly suggesting a depletion of OT. There is also evidence that chronic exposure to elevated VP, which may occur in some psychedelics, can downregulate OT receptors [212]. OT generally has calming or therapeutic consequences [17], while the lasting effects of VP are dynamic and not well documented.

## 12 Conclusions and hypothesis

The literature described in this review suggests the hypothesis that knowledge of the properties of the VP-OT system [17,56,193] may help to explain apparently paradoxical effects in reaction to psychedelics. Awareness of these interactions also may help to predict or avoid physical and emotional dangers sometimes associated with psychedelic use [138,139]. However, the VP-OT system is dynamic and difficult to study, in part due to the capacity of these peptides to influence each other's receptors, with changes that occur across time.

The hypotheses described here are for the most part based on correlational observations and fragments of knowledge. With those caveats in mind, I suggested that VP, with interactions with other molecules such as serotonin, cytokines, dopamine and opioids, could set a physiological stage upon which OT acts. This in turn could have broad benefits to mammalian physiology (Fig. 1). However, the evolved relationship between VP and OT may require that both peptides be initially active to maximize the benefits of OT or psychedelics; this hypothesis remains untested.

The VP-OT interaction is of particular relevance in the management of **challenges and stressors in a social context and over time.** For example, OT and VP and their receptors are involved in the regulation of emotional context, homeostasis and allostasis [213]. OT also plays a central role in the protective effects of sociality during stressful experiences, and is a critical component of the physiology we have termed “sociostasis” [193].

The recreational use of psychedelics may be intended to be transitory. However, medical applications of psychedelics are typically sought for their long-term/chronic benefits. Epigenetic consequences of psychedelics also are plausible, especially when drug effects are long-lasting [214], although these have only recently begun to receive attention [122]. For example, neural plasticity may be viewed as of value in reducing treatment-resistant depression, dealing with intrusive memories or creating openness to other therapies and new relationships. Thus, plasticity is often described as a beneficial consequence of some psychedelics, especially in depressed patients [215,216]. However, neural plasticity also carries unpredictable risks, such as the loss of desired memories [191].

An emerging goal in contemporary psychiatry and pharmacology has been to prevent or reverse the negative consequences of stress or trauma, including depression, anxiety or neurodegeneration [217]. Clinical research has focused on the potential of psychedelics to beneficially influence reactivity to various kinds of challenges. However, for some individuals the use of psychedelics also can present benefits that may be overestimated due to expectations [218].

I propose that studies of interactions such as those described here will be necessary to create a deeper understanding of the actions of psychedelics. These interactions also suggest a novel perspective on the general role of neuropeptides, including VP and OT, in stress-related and time-dependent disorders and the processes that are called hormesis (Table 2) [37,200].

## Funding

My research studies have been repeatedly funded by the National Institute of Health and other agencies and foundations. But that funding was not directly relevant to this review.

## Declaration of Competing Interest

The author declares that they have no known competing financial interests or personal relationships that could have appeared to influence the work reported in this paper.

The author is an Editorial Board Member for Comprehensive Psychoneuroendocrinology and was not involved in the editorial review or the decision to publish this article.

## References

[1] Goodwin R.D., Dierker L.C., Wu M., Galea S., Hoven C.W., Weinberger A.H. (2022). Trends in U.S. Depression prevalence from 2015 to 2020: the widening treatment gap. Am. J. Prev. Med..

[2] Aguilera G. (2011). Regulation of the hypothalamic-pituitary-adrenal axis by neuropeptides. Horm. Mol. Biol. Clin. Invest..

[3] Caruso A., Gaetano A., Scaccianoce S. (2022). Corticotropin-releasing hormone: biology and therapeutic opportunities. Biology (Basel).

[4] Holsboer F., Ising M. (2024). Precision psychiatry approach to treat depression and anxiety targeting the stress hormone system—V1b-antagonists as a case in point. Pharmacopsychiatry.

[5] Heifets B.D., Salgado J.S., Taylor M.D., Hoerbelt P., Cardozo Pinto D.F., Steinberg E.E., Walsh J.J., Sze J.Y., Malenka R.C. (2019). Distinct neural mechanisms for the prosocial and rewarding properties of MDMA. Sci. Transl. Med..

[6] Muir J., Lin S., Aarrestad I.K., Daniels H.R., Ma J., Tian L., Olson D.E., Kim C.K. (2024). Isolation of psychedelic-responsive neurons underlying anxiolytic behavioral states. Science (New York, N.Y.).

[7] Raison C.L., Sanacora G., Woolley J., Heinzerling K., Dunlop B.W., Brown R.T., Kakar R., Hassman M., Trivedi R.P., Robison R., Gukasyan N., Nayak S.M., Hu X., O'Donnell K.C., Kelmendi B., Sloshower J., Penn A.D., Bradley E., Kelly D.F., Griffiths R.R. (2023). Single-dose psilocybin treatment for major depressive disorder: a randomized clinical trial. JAMA.

[8] Ramaekers J.G., Reckweg J.T., Mason N.L. (2025). Benefits and challenges of ultra-fast, short-acting psychedelics in the treatment of depression. Am. J. Psychiatr..

[9] Saeger H.N., Olson D.E. (2022). Psychedelic-inspired approaches for treating neurodegenerative disorders. J. Neurochem..

[10] Zaretsky T.G., Jagodnik K.M., Barsic R., Antonio J.H., Bonanno P.A., MacLeod C., Pierce C., Carney H., Morrison M.T., Saylor C., Danias G., Lepow L., Yehuda R. (2024). The psychedelic future of post-traumatic stress disorder treatment. Curr. Neuropharmacol..

[11] Brouwer A., Brown J.K., Erowid E., Erowid F., Thyssen S., Raison C.L., Carhart-Harris R.L. (2025). A qualitative analysis of the psychedelic mushroom come-up and come-down. Npj Mental Health Research.

[12] Nardou R., Sawyer E., Song Y.J., Wilkinson M., Padovan-Hernandez Y., de Deus J.L., Wright N., Lama C., Faltin S., Goff L.A., Stein-O’Brien G.L., Dölen G. (2023). Psychedelics reopen the social reward learning critical period. Nature.

[13] van Elk M., Yaden D.B. (2022). Pharmacological, neural, and psychological mechanisms underlying psychedelics: a critical review. Neurosci. Biobehav. Rev..

[14] Bonnelle V., Feilding A., Rosas F.E., Nutt D.J., Carhart-Harris R.L., Timmermann C. (2024).

[15] Nutt D., Erritzoe D., Carhart-Harris R. (2020). Psychedelic psychiatry's brave new world. Cell.

[16] Nardou R., Lewis E.M., Rothhaas R., Xu R., Yang A., Boyden E., Dölen G. (2019). Oxytocin-dependent reopening of a social reward learning critical period with MDMA. Nature.

[17] Carter C.S., Kenkel W.M., MacLean E.L., Wilson S.R., Perkeybile A.M., Yee J.R., Ferris C.F., Nazarloo H.P., Porges S.W., Davis J.M., Connelly J.J., Kingsbury M.A. (2020). Is oxytocin “nature's medicine”. Pharmacol. Rev..

[18] Carhart-Harris R.L., Chandaria S., Erritzoe D.E., Gazzaley A., Girn M., Kettner H., Mediano P.a.M., Nutt D.J., Rosas F.E., Roseman L., Timmermann C., Weiss B., Zeifman R.J., Friston K.J. (2023). Canalization and plasticity in psychopathology. Neuropharmacology.

[19] Carter C.S., Kingsbury M.A. (2022). Oxytocin and oxygen: the evolution of a solution to the “stress of life.”. Phil. Trans. Roy. Soc. Lond. B Biol. Sci..

[20] Baumeister D., Ciufolini S., Mondelli V. (2016). Effects of psychotropic drugs on inflammation: consequence or mediator of therapeutic effects in psychiatric treatment?. Psychopharmacology.

[21] Flanagan T.W., Nichols C.D. (2022). Psychedelics and anti-inflammatory activity in animal models. Curr. Topics Behavio. Neurosci..

[22] Nikkheslat N. (2021). Targeting inflammation in depression: ketamine as an anti-inflammatory antidepressant in psychiatric emergency. Brain, Behavior Immunity - Health.

[23] Ferris C.F. (2008). Functional magnetic resonance imaging and the neurobiology of vasopressin and oxytocin. Prog. Brain Res..

[24] Atila C., Refardt J., Christ-Crain M. (2024). Arginine vasopressin deficiency: diagnosis, management and the relevance of oxytocin deficiency. Nat. Rev. Endocrinol..

[25] Landgraf R., Neumann I.D. (2004). Vasopressin and oxytocin release within the brain: a dynamic concept of multiple and variable modes of neuropeptide communication. Front. Neuroendocrinol..

[26] Makwana R., Crawley E., Straface M., Palmer A., Gharibans A., Devalia K., Loy J., O'Grady G., Andrews P.L.R., Sanger G.J. (2022). Synergistic augmentation of rhythmic myogenic contractions of human stomach by arginine vasopressin and adrenaline: implications for the induction of nausea. Br. J. Pharmacol..

[27] Breeksema J.J., Kuin B.W., Kamphuis J., van den Brink W., Vermetten E., Schoevers R.A. (2022). Adverse events in clinical treatments with serotonergic psychedelics and MDMA: a mixed-methods systematic review. J. Psychopharmacol..

[28] Cue L., Chu F., Cascella M. (2025). StatPearls.

[29] Dantzer R. (2021). Love and fear in the times of sickness. Comprehen. Psychoneuroendocrinol..

[30] Hart B.L. (1988). Biological basis of the behavior of sick animals. Neurosci. Biobehav. Rev..

[31] Bao A.-M., Meynen G., Swaab D.F. (2008). The stress system in depression and neurodegeneration: focus on the human hypothalamus. Brain Res. Rev..

[32] Chrousos G.P. (2009). Stress and disorders of the stress system. Nat. Rev. Endocrinol..

[33] Selye H. (1956).

[34] Selye H. (1975). Confusion and controversy in the stress field. J. Hum. Stress.

[35] Casaril A.M., Dantzer R., Bas-Orth C. (2021). Neuronal mitochondrial dysfunction and bioenergetic failure in inflammation-associated depression. Front. Neurosci..

[36] Dantzer R. (2022). Evolutionary aspects of infections: inflammation and sickness behaviors. Curr. Topics Behavio. Neurosci..

[37] Calabrese E.J., Mattson M.P. (2024). The catabolic—anabolic cycling hormesis model of health and resilience. Ageing Res. Rev..

[38] Mattson M.P., Leak R.K. (2024). The hormesis principle of neuroplasticity and neuroprotection. Cell Metab..

[39] Bowen M.T., McGregor I.S. (2014). Oxytocin and vasopressin modulate the social response to threat: a preclinical study. Int. J. Neuropsychopharmacol..

[40] Dölen G., Darvishzadeh A., Huang K.W., Malenka R.C. (2013). Social reward requires coordinated activity of nucleus accumbens oxytocin and serotonin. Nature.

[41] Holze F., Avedisian I., Varghese N., Eckert A., Liechti M.E. (2021). Role of the 5-ht2a receptor in acute effects of LSD on empathy and circulating oxytocin. Front. Pharmacol..

[42] Bao A.-M., Swaab D.F. (2019). The human hypothalamus in mood disorders: the HPA axis in the center. IBRO Reports.

[43] Armstrong W.E., Foehring R.C., Kirchner M.K., Sladek C.D. (2019). Electrophysiological properties of identified oxytocin and vasopressin neurones. J. Neuroendocrinol..

[44] Carter C.S. (2014). Oxytocin pathways and the evolution of human behavior. Annu. Rev. Psychol..

[45] Knobloch H.S., Grinevich V. (2014). Evolution of oxytocin pathways in the brain of vertebrates. Front. Behav. Neurosci..

[46] Sartorius A.M., Rokicki J., Birkeland S., Bettella F., Barth C., de Lange A.-M.G., Haram M., Shadrin A., Winterton A., Steen N.E., Schwarz E., Stein D.J., Andreassen O.A., van der Meer D., Westlye L.T., Theofanopoulou C., Quintana D.S. (2024). An evolutionary timeline of the oxytocin signaling pathway. Commun. Biol..

[47] Caldwell H.K. (2017). Oxytocin and vasopressin: powerful regulators of social behavior. Neuroscientist.

[48] Song Z., Borland J.M., Larkin T.E., O'Malley M., Albers H.E. (2016). Activation of oxytocin receptors, but not arginine-vasopressin V1a receptors, in the ventral tegmental area of male Syrian hamsters is essential for the reward-like properties of social interactions. Psychoneuroendocrinology.

[49] Wronikowska-Denysiuk O., Mrozek W., Budzyńska B. (2023). The role of oxytocin and vasopressin in drug-induced reward-implications for social and non-social factors. Biomolecules.

[50] Moberg K.U. (2024). Oxytocin in growth, reproduction, restoration and health. Comprehen. Psychoneuroendocrinol..

[51] Neumann I.D. (2002). Involvement of the brain oxytocin system in stress coping: interactions with the hypothalamo-pituitary-adrenal axis. Prog. Brain Res..

[52] Antoni F.A. (2019). Magnocellular vasopressin and the mechanism of “glucocorticoid escape.”. Front. Endocrinol..

[53] Goh K.K., Chen C.-H., Lane H.-Y. (2021). Oxytocin in schizophrenia: pathophysiology and implications for future treatment. Int. J. Mol. Sci..

[54] Ronan P.J., Korzan W.J., Johnson P.L., Lowry C.A., Renner K.J., Summers C.H. (2023). Prior stress and vasopressin promote corticotropin-releasing factor inhibition of serotonin release in the central nucleus of the amygdala. Front. Behav. Neurosci..

[55] Shen X.Z., Li Y., Li L., Shah K.H., Bernstein K.E., Lyden P., Shi P. (2015). Microglia participate in neurogenic regulation of hypertension. Hypertension.

[56] Ellis B.J., Horn A.J., Carter C.S., van IJzendoorn M.H., Bakermans-Kranenburg M.J. (2021). Developmental programming of oxytocin through variation in early-life stress: four meta-analyses and a theoretical reinterpretation. Clin. Psychol. Rev..

[57] Kompier N.F., Keysers C., Gazzola V., Lucassen P.J., Krugers H.J. (2019). Early life adversity and adult social behavior: focus on arginine vasopressin and oxytocin as potential mediators. Front. Behav. Neurosci..

[58] Hu H., Zarate C.A., Verbalis J. (2024). Arginine vasopressin in mood disorders: a potential biomarker of disease pathology and a target for pharmacologic intervention. Psychiatr. Clin. Neurosci..

[59] Horn A.J., Cole S., Nazarloo H.P., Nazarloo P., Davis J.M., Carrier D., Bryan C., Carter C.S. (2024). Severe PTSD is marked by reduced oxytocin and elevated vasopressin. Comprehen. Psychoneuroendocrinol..

[60] Aulinas A., Lawson E.A. (2025). The oxytocin system and implications for oxytocin deficiency in hypothalamic-pituitary disease. Endocr. Rev..

[61] Carter C.S. (2022). Oxytocin and love: myths, metaphors and mysteries. Comprehen. Psychoneuroendocrinol..

[62] Feldman R. (2020). What is resilience: an affiliative neuroscience approach. World Psychiatry: Offic. J. World Psychiatric Assoc..

[63] Janeček M., Dabrowska J. (2019). Oxytocin facilitates adaptive fear and attenuates anxiety responses in animal models and human studies-potential interaction with the corticotropin-releasing factor (CRF) system in the bed nucleus of the stria terminalis (BNST). Cell Tissue Res..

[64] Carter C.S. (2003). Developmental consequences of oxytocin. Physiol. Behav..

[65] Froemke R.C., Young L.J. (2021). Oxytocin, neural plasticity, and social behavior. Annu. Rev. Neurosci..

[66] Pekarek B.T., Kochukov M., Lozzi B., Wu T., Hunt P.J., Tepe B., Hanson Moss E., Tantry E.K., Swanson J.L., Dooling S.W., Patel M., Belfort B.D.W., Romero J.M., Bao S., Hill M.C., Arenkiel B.R. (2022). Oxytocin signaling is necessary for synaptic maturation of adult-born neurons. Gene Dev..

[67] Paquin J., Danalache B.A., Jankowski M., McCann S.M., Gutkowska J. (2002). Oxytocin induces differentiation of P19 embryonic stem cells to cardiomyocytes. Proc. Natl. Acad. Sci. U. S. A.

[68] Leuner B., Caponiti J.M., Gould E. (2012). Oxytocin stimulates adult neurogenesis even under conditions of stress and elevated glucocorticoids. Hippocampus (New York, N. Y.).

[69] Meinung C.-P., Boi L., Pandamooz S., Mazaud D., Ghézali G., Rouach N., Neumann I.D. (2024). OXTR-mediated signaling in astrocytes contributes to anxiolysis. Mol. Psychiatr..

[70] Turkin A., Tuchina O., Klempin F. (2021). Microglia function on precursor cells in the adult Hippocampus and their responsiveness to serotonin signaling. Front. Cell Dev. Biol..

[71] Wahis J., Baudon A., Althammer F., Kerspern D., Goyon S., Hagiwara D., Lefevre A., Barteczko L., Boury-Jamot B., Bellanger B., Abatis M., Da Silva Gouveia M., Benusiglio D., Eliava M., Rozov A., Weinsanto I., Knobloch-Bollmann H.S., Kirchner M.K., Roy R.K., Charlet A. (2021). Astrocytes mediate the effect of oxytocin in the central amygdala on neuronal activity and affective states in rodents. Nat. Neurosci..

[72] Xiao S., Fischer H., Ebner N.C., Rukh G., Dang J., Westberg L., Schiöth H.B. (2024). Oxytocin pathway gene variation and corticostriatal resting-state functional connectivity. Comprehen. Psychoneuroendocrinol..

[73] Bordt E.A., Ceasrine A.M., Bilbo S.D. (2020). Microglia and sexual differentiation of the developing brain: a focus on ontogeny and intrinsic factors. GLIA (New York, N. Y.).

[74] Yee J.R., Kenkel W.M., Frijling J.L., Dodhia S., Onishi K.G., Tovar S., Saber M.J., Lewis G.F., Liu W., Porges S.W., Carter C.S. (2016). Oxytocin promotes functional coupling between paraventricular nucleus and both sympathetic and parasympathetic cardioregulatory nuclei. Horm. Behav..

[75] Maroun M., Sarussi-Elyahu A., Yaseen A., Hatoum O.A., Kritman M. (2020). Sex-dimorphic role of prefrontal oxytocin receptors in social-induced facilitation of extinction in juvenile rats. Transl. Psychiatry.

[76] Sripada C.S., Phan K.L., Labuschagne I., Welsh R., Nathan P.J., Wood A.G. (2013). Oxytocin enhances resting-state connectivity between amygdala and medial frontal cortex. Int. J. Neuropsychopharmacol..

[77] Carter C.S. (2023). Close encounters with oxytocin. Comprehen. Psychoneuroendocrinol..

[78] Carter C.S. (1992). Oxytocin and sexual behavior. Neurosci. Biobehav. Rev..

[79] Arrowsmith S., Wray S. (2014). Oxytocin: its mechanism of action and receptor signalling in the myometrium. J. Neuroendocrinol..

[80] Liberzon I., Chalmers D.T., Mansour A., Lopez J.F., Watson S.J., Young E.A. (1994). Glucocorticoid regulation of hippocampal oxytocin receptor binding. Brain Res..

[81] Ben-Ari Y. (2015). Is birth a critical period in the pathogenesis of autism spectrum disorders?. Nat. Rev. Neurosci..

[82] Thompson M.R., Hunt G.E., McGregor I.S. (2009). Neural correlates of MDMA (“Ecstasy”)-induced social interaction in rats. Soc. Neurosci..

[83] Danoff J.S., Connelly J.J., Morris J.P., Perkeybile A.M. (2021). An epigenetic rheostat of experience: DNA methylation of OXTR as a mechanism of early life allostasis. Comprehen. Psychoneuroendocrinol..

[84] Jurek B., Neumann I.D. (2018). The oxytocin receptor: from intracellular signaling to behavior. Physiol. Rev..

[85] Kanes S.J., Dennie L., Perera P. (2023). Targeting the arginine vasopressin V1b receptor system and stress response in depression and other neuropsychiatric disorders. Neuropsychiatric Dis. Treat..

[86] Chini B., Leonzino M., Braida D., Sala M. (2014). Learning about oxytocin: pharmacologic and behavioral issues. Biol. Psychiatry.

[87] Smith C.J.W., DiBenedictis B.T., Veenema A.H. (2019). Comparing vasopressin and oxytocin fiber and receptor density patterns in the social behavior neural network: implications for cross-system signaling. Front. Neuroendocrinol..

[88] Francesconi W., Olivera-Pasilio V., Berton F., Olson S.L., Chudoba R., Monroy L.M., Krabichler Q., Grinevich V., Dabrowska J. (2024). Like sisters but not twins—vasopressin and oxytocin excite BNST neurons via cell type-specific expression of oxytocin receptor to reduce anxious arousal. bioRxiv: Preprint Server Biol..

[89] Rigney N., Whylings J., de Vries G.J., Petrulis A. (2021). Sex differences in the control of social investigation and anxiety by vasopressin cells of the paraventricular nucleus of the hypothalamus. Neuroendocrinology (Basel).

[90] Carter C.S. (2017). The oxytocin-vasopressin pathway in the context of love and fear. Front. Endocrinol..

[91] Althammer F., Eliava M., Grinevich V. (2021). Central and peripheral release of oxytocin: relevance of neuroendocrine and neurotransmitter actions for physiology and behavior. Handb. Clin. Neurol..

[92] Welch M.G., Tamir H., Gross K.J., Chen J., Anwar M., Gershon M.D. (2009). Expression and developmental regulation of oxytocin (OT) and oxytocin receptors (OTR) in the enteric nervous system (ENS) and intestinal epithelium. J. Comp. Neurol..

[93] Ostrowski N.L., Lolait S.J., Bradley D.J., O'Carroll A.M., Brownstein M.J., Young W.S. (1992). Distribution of V1a and V2 vasopressin receptor messenger ribonucleic acids in rat liver, kidney, pituitary and brain. Endocrinology.

[94] Kingsbury M.A. (2024). The intertwining of oxytocin's effects on social affiliation and inflammation. Comprehen. Psychoneuroendocrinol..

[95] Jurek B., Meyer M. (2020). Anxiolytic and anxiogenic? How the transcription factor MEF2 might explain the manifold behavioral effects of oxytocin. Front. Endocrinol..

[96] Wei J., Zheng H., Li G., Chen Z., Fang G., Yan J. (2023). Involvement of oxytocin receptor deficiency in psychiatric disorders and behavioral abnormalities. Front. Cell. Neurosci..

[97] Vaccari C., Lolait S.J., Ostrowski N.L. (1998). Comparative distribution of vasopressin V1b and oxytocin receptor messenger ribonucleic acids in brain. Endocrinology.

[98] Williams Avram S.K., Lee H.-J., Fastman J., Cymerblit-Sabba A., Smith A., Vincent M., Song J., Granovetter M.C., Lee S.-H., Cilz N.I., Stackmann M., Chaturvedi R., Young W.S. (2019). NMDA receptor in vasopressin 1b neurons is not required for short-term social memory, object memory or aggression. Front. Behav. Neurosci..

[99] Kim M.S., Chey W.D., Owyang C., Hasler W.L. (1997). Role of plasma vasopressin as a mediator of nausea and gastric slow wave dysrhythmias in motion sickness. Am. J. Physiol..

[100] Amato S., Averna M., Guidolin D., Ceccoli C., Gatta E., Candiani S., Pedrazzi M., Capraro M., Maura G., Agnati L.F., Cervetto C., Marcoli M. (2023). Heteromerization of dopamine D2 and oxytocin receptor in adult striatal astrocytes. Int. J. Mol. Sci..

[101] Borroto-Escuela D.O., Cuesta-Marti C., Lopez-Salas A., Chruścicka-Smaga B., Crespo-Ramírez M., Tesoro-Cruz E., Palacios-Lagunas D.A., Perez de la Mora M., Schellekens H., Fuxe K. (2022). The oxytocin receptor represents a key hub in the GPCR heteroreceptor network: potential relevance for brain and behavior. Front. Mol. Neurosci..

[102] Mustafa N.S., Bakar N.H.A., Mohamad N., Adnan L.H.M., Fauzi N.F.A.M., Thoarlim A., Omar S.H.S., Hamzah M.S., Yusoff Z., Jufri M., Ahmad R. (2020). MDMA and the brain: a short review on the role of neurotransmitters in neurotoxicity. Basic Clin. Neurosci..

[103] Jørgensen H., Riis M., Knigge U., Kjaer A., Warberg J. (2003). Serotonin receptors involved in vasopressin and oxytocin secretion. J. Neuroendocrinol..

[104] Soslau G. (2022). Cardiovascular serotonergic system: evolution, receptors, transporter, and function. J. Exp. Zool. Part A: Ecological and Integrative Physiology.

[105] Marazziti D., Diep P.-T., Carter S., Carbone M.G. (2022). Oxytocin: an old hormone, a novel psychotropic drug and its possible use in treating psychiatric disorders. Curr. Med. Chem..

[106] Patel T.N., Caiola H.O., Mallari O.G., Blandino K.L., Goldenthal A.R., Dymecki S.M., Rood B.D. (2022). Social interactions increase activation of vasopressin-responsive neurons in the dorsal raphe. Neuroscience.

[107] Mitroshina E.V., Marasanova E.A., Vedunova M.V. (2023). Functional dimerization of serotonin receptors: role in health and depressive disorders. Int. J. Mol. Sci..

[108] Ismaylova E., Nemoda Z., Booij L. (2025). Brain serotonin, oxytocin, and their interaction: relevance for eating disorders. J. Psychopharmacol..

[109] Lefevre A., Richard N., Jazayeri M., Beuriat P.-A., Fieux S., Zimmer L., Duhamel J.-R., Sirigu A. (2017). Oxytocin and serotonin brain mechanisms in the nonhuman primate. J. Neurosci..

[110] Mottolese R., Redouté J., Costes N., Le Bars D., Sirigu A. (2014). Switching brain serotonin with oxytocin. Proc. Natl. Acad. Sci. U. S. A.

[111] Yoshida M., Takayanagi Y., Inoue K., Kimura T., Young L.J., Onaka T., Nishimori K. (2009). Evidence that oxytocin exerts anxiolytic effects via oxytocin receptor expressed in serotonergic neurons in mice. J. Neurosci..

[112] Javed A., Kamradt M.C., Van de Kar L.D., Gray T.S. (1999). D-Fenfluramine induces serotonin-mediated Fos expression in corticotropin-releasing factor and oxytocin neurons of the hypothalamus, and serotonin-independent Fos expression in enkephalin and neurotensin neurons of the amygdala. Neuroscience.

[113] Levin G., Ein-Dor T. (2023). A unified model of the biology of peripartum depression. Transl. Psychiatry.

[114] Humble M.B., Bejerot S. (2016). Orgasm, serotonin reuptake inhibition, and plasma oxytocin in obsessive-compulsive disorder. Gleaning from a distant randomized clinical trial. Sex. Med..

[115] Ferris C. (1992). Role of vasopressin in aggressive and dominant/subordinate behaviors. Ann. N. Y. Acad. Sci..

[116] Erritzoe D., Barba T., Spriggs M.J., Rosas F.E., Nutt D.J., Carhart-Harris R. (2024). Effects of discontinuation of serotonergic antidepressants prior to psilocybin therapy versus escitalopram for major depression. J. Psychopharmacol..

[117] Gabriel M., Sharma V. (2017). Antidepressant discontinuation syndrome. CMAJ (Can. Med. Assoc. J.) : Can. Med. Assoc. J..

[118] Mehdi S.F., Pusapati S., Khenhrani R.R., Farooqi M.S., Sarwar S., Alnasarat A., Mathur N., Metz C.N., LeRoith D., Tracey K.J., Yang H., Brownstein M.J., Roth J. (2022). Oxytocin and related peptide hormones: candidate anti-inflammatory therapy in early stages of sepsis. Front. Immunol..

[119] Shapiro B., Kramer E., Khoury D., Preda A. (2023). Establishing core symptoms of acute serotonin reuptake inhibitor withdrawal: results from an international survey of online peer-support communities. Pharmacopsychiatry.

[120] Sharp T., Collins H. (2023). Mechanisms of SSRI therapy and discontinuation. Curr. Topics Behavio. Neurosci..

[121] Price C.M., Feduccia A.A., DeBonis K. (2022). Effects of selective serotonin reuptake inhibitor use on 3,4-methylenedioxymethamphetamine-assisted therapy for posttraumatic stress disorder: a review of the evidence, neurobiological plausibility, and clinical significance. J. Clin. Psychopharmacol..

[122] Acero V.P., Cribas E.S., Browne K.D., Rivellini O., Burrell J.C., O'Donnell J.C., Das S., Cullen D.K. (2023). Bedside to bench: the outlook for psychedelic research. Front. Pharmacol..

[123] Kim Y.-K., Kim O.Y., Song J. (2020). Alleviation of depression by glucagon-like peptide 1 through the regulation of neuroinflammation, neurotransmitters, neurogenesis, and synaptic function. Front. Pharmacol..

[124] Leon R.M., Borner T., Reiner D.J., Stein L.M., Lhamo R., De Jonghe B.C., Hayes M.R. (2019). Hypophagia induced by hindbrain serotonin is mediated through central GLP-1 signaling and involves 5-HT2C and 5-HT3 receptor activation. Neuropsychopharmacology.

[125] Daskalakis N.P., Bagot R.C., Parker K.J., Vinkers C.H., de Kloet E.R. (2013). The three-hit concept of vulnerability and resilience: towards understanding adaptation to early-life adversity outcome. Psychoneuroendocrinology.

[126] Gordon J.A., Hen R. (2004). The serotonergic system and anxiety. NeuroMolecular Med..

[127] Vamvakopoulou I.A., Nutt D.J. (2024). Psychedelics: from cave art to 21st-century medicine for addiction. Eur. Addctn. Res..

[128] Bouso J.C., Andión Ó., Sarris J.J., Scheidegger M., Tófoli L.F., Opaleye E.S., Schubert V., Perkins D. (2022). Adverse effects of ayahuasca: results from the global ayahuasca survey. PLOS Global Public Health.

[129] Vogt S.B., Ley L., Erne L., Straumann I., Becker A.M., Klaiber A., Holze F., Vandersmissen A., Mueller L., Duthaler U., Rudin D., Luethi D., Varghese N., Eckert A., Liechti M.E. (2023). Acute effects of intravenous DMT in a randomized placebo-controlled study in healthy participants. Transl. Psychiatry.

[130] Banushi B., Polito V. (2023). A comprehensive review of the current status of the cellular neurobiology of psychedelics. Biology (Basel).

[131] Johnson M.W., Hendricks P.S., Barrett F.S., Griffiths R.R. (2019). Classic psychedelics: an integrative review of epidemiology, therapeutics, mystical experience, and brain network function. Pharmacol. Therapeut..

[132] Nichols D.E. (2016). Psychedelics. Pharmacol. Rev..

[133] Molla H., Lee R., Lyubomirsky S., de Wit H. (2023). Drug-induced social connection: both MDMA and methamphetamine increase feelings of connectedness during controlled dyadic conversations. Sci. Rep..

[134] Nichols D.E. (2022). Entactogens: how the name for a novel class of psychoactive agents originated. Front. Psychiatr..

[135] Mash D.C. (2023). Iuphar - invited review—Ibogaine—A legacy within the current renaissance of psychedelic therapy. Pharmacol. Res..

[136] Ermakova A.O., Dunbar F., Rucker J., Johnson M.W. (2022). A narrative synthesis of research with 5-MeO-DMT. J. Psychopharmacol..

[137] Kuypers K.P.C., de la Torre R., Farre M., Pizarro N., Xicota L., Ramaekers J.G. (2018). MDMA-induced indifference to negative sounds is mediated by the 5-HT2A receptor. Psychopharmacology.

[138] Schmid Y., Enzler F., Gasser P., Grouzmann E., Preller K.H., Vollenweider F.X., Brenneisen R., Müller F., Borgwardt S., Liechti M.E. (2015). Acute effects of lysergic acid diethylamide in healthy subjects. Biol. Psychiatry.

[139] Zeifman R.J., Kettner H., Pagni B.A., Mallard A., Roberts D.E., Erritzoe D., Ross S., Carhart-Harris R.L. (2023). Co-use of MDMA with psilocybin/LSD may buffer against challenging experiences and enhance positive experiences. Sci. Rep..

[140] Liechti M.E., Saur M.R., Gamma A., Hell D., Vollenweider F.X. (2000). Psychological and physiological effects of MDMA (“Ecstasy”) after pretreatment with the 5-HT(2) antagonist ketanserin in healthy humans. Neuropsychopharmacology: Offi. Public. Am. College Neuropsychopharmacol..

[141] Edem E.E., Oguntala O.A., Ikuelogbon D.A., Nebo K.E., Fafure A.A., Akinluyi E.T., Isaac G.T., Kunlere O.E. (2023). Prolonged ketamine therapy differentially rescues psychobehavioural deficits via modulation of nitro-oxidative stress and oxytocin receptors in the gut-brain-axis of chronically-stressed mice. Psychoneuroendocrinology.

[142] Krystal J.H., Abdallah C.G., Sanacora G., Charney D.S., Duman R.S. (2019). Ketamine: a paradigm shift for depression research and treatment. Neuron (Camb., Mass.).

[143] Denomme N., Heifets B.D. (2024). Ketamine, the first associative anesthetic? Some considerations on classifying psychedelics, entactogens, and dissociatives. Am. J. Psychiatr..

[144] Sleigh J., Harvey M., Voss L., Denny B. (2014). Ketamine – more mechanisms of action than just NMDA blockade. Trends Anaesthe. Critical Care.

[145] Yamamoto S., Ohba H., Nishiyama S., Harada N., Kakiuchi T., Tsukada H., Domino E.F. (2013). Subanesthetic doses of ketamine transiently decrease serotonin transporter activity: a pet study in conscious monkeys. Neuropsychopharmacology.

[146] Tiger M., Veldman E.R., Ekman C.-J., Halldin C., Svenningsson P., Lundberg J. (2020). A randomized placebo-controlled PET study of ketamine's effect on serotonin1B receptor binding in patients with SSRI-resistant depression. Transl. Psychiatry.

[147] Hess E.M., Greenstein D.K., Hutchinson O.L., Zarate C.A., Gould T.D. (2024). Entactogen effects of ketamine: a reverse-translational study. Am. J. Psychiatr..

[148] Huang M.-C., Chen L.-Y., Chang H.-M., Liang X.-Y., Chen C.-K., Cheng W.-J., Xu K. (2018). Decreased blood levels of oxytocin in ketamine-dependent patients during early abstinence. Front. Psychiatr..

[149] Mawere-Mubvumbi T.P. (2023). S-ketamine: is it a ride worth taking? Adverse effects associated with S-ketamine use as an adjuvant or single agent drug. Trends Anaesthe. Critical Care.

[150] McGregor I.S., Callaghan P.D., Hunt G.E. (2008). From ultrasocial to antisocial: a role for oxytocin in the acute reinforcing effects and long-term adverse consequences of drug use?. Br. J. Pharmacol..

[151] Yue Y., Wan X., Liu G., Zhu T., Xu D., Zhao M., Cai Y., Murayama R., Hashimoto H., Anzai N., Hashimoto K. (2025). Subdiaphragmatic vagotomy reduces hypothalamic oxytocin expression and blood levels after oral MDMA administration in male rats. Prog. Neuro Psychopharmacol. Biol. Psychiatr..

[152] De Gregorio D., Inserra A., Enns J.P., Markopoulos A., Pileggi M., El Rahimy Y., Lopez-Canul M., Comai S., Gobbi G. (2022). Repeated lysergic acid diethylamide (LSD) reverses stress-induced anxiety-like behavior, cortical synaptogenesis deficits and serotonergic neurotransmission decline. Neuropsychopharmacology.

[153] Ley L., Holze F., Arikci D., Becker A.M., Straumann I., Klaiber A., Coviello F., Dierbach S., Thomann J., Duthaler U., Luethi D., Varghese N., Eckert A., Liechti M.E. (2023). Comparative acute effects of mescaline, lysergic acid diethylamide, and psilocybin in a randomized, double-blind, placebo-controlled cross-over study in healthy participants. Neuropsychopharmacology: Offi. Public. Am. College Neuropsychopharmacol..

[154] Constantino J.L., van Dalfsen J.H., Massetti S., Kamphuis J., Schoevers R.A. (2025). Neurobiological mechanisms of antidepressant properties of psilocybin: a systematic review of blood biomarkers. Prog. Neuro Psychopharmacol. Biol. Psychiatr..

[155] Forsling M.L., Fallon J.K., Shah D., Tilbrook G.S., Cowan D.A., Kicman A.T., Hutt A.J. (2002). The effect of 3,4-methylenedioxymethamphetamine (MDMA, ?ecstasy?) and its metabolites on neurohypophysial hormone release from the isolated rat hypothalamus. Br. J. Pharmacol..

[156] Henry J.A., Fallon J.K., Kicman A.T., Hutt A.J., Cowan D.A., Forsling M. (1998). Low-dose MDMA (“ecstasy”) induces vasopressin secretion. Lancet (London, England).

[157] Atila C., Holze F., Murugesu R., Rommers N., Hutter N., Varghese N., Sailer C.O., Eckert A., Heinrichs M., Liechti M.E., Christ-Crain M. (2023). Oxytocin in response to MDMA provocation test in patients with arginine vasopressin deficiency (central diabetes insipidus): a single-centre, case-control study with nested, randomised, double-blind, placebo-controlled crossover trial. Lancet Diabetes Endocrinol..

[158] Fabian M., Forsling M.L., Jones J.J., Pryor J.S. (1969). The clearance and antidiuretic potency of neurohypophysial hormones in man, and their plasma binding and stability. J. Physiol. (Camb.).

[159] Ferris C.F. (2008). Functional magnetic resonance imaging and the neurobiology of vasopressin and oxytocin. Prog. Brain Res..

[160] Goh K.K., Chen C.Y.-A., Wu T.-H., Chen C.-H., Lu M.-L. (2022). Crosstalk between schizophrenia and metabolic syndrome: the role of oxytocinergic dysfunction. Int. J. Mol. Sci..

[161] Goh K.K., Lu M.-L. (2022). Relationship between the domains of theory of mind, social dysfunction, and oxytocin in schizophrenia. J. Psychiatr. Res..

[162] Mitchell J.M., Ot’alora G.M., van der Kolk B., Shannon S., Bogenschutz M., Gelfand Y., Paleos C., Nicholas C.R., Quevedo S., Balliett B., Hamilton S., Mithoefer M., Kleiman S., Parker-Guilbert K., Tzarfaty K., Harrison C., de Boer A., Doblin R., Yazar-Klosinski B. (2023). MDMA-assisted therapy for moderate to severe PTSD: a randomized, placebo-controlled phase 3 trial. Nat. Med..

[163] Olff M., Frijling J.L., Kubzansky L.D., Bradley B., Ellenbogen M.A., Cardoso C., Bartz J.A., Yee J.R., van Zuiden M. (2013). The role of oxytocin in social bonding, stress regulation and mental health: an update on the moderating effects of context and interindividual differences. Psychoneuroendocrinology.

[164] Porges S.W. (2021). Polyvagal Theory: a biobehavioral journey to sociality. Comprehen. Psychoneuroendocrinol..

[165] De Filippo R., Schmitz D. (2024). Synthetic surprise as the foundation of the psychedelic experience. Neurosci. Biobehav. Rev..

[166] Gattuso J.J., Perkins D., Ruffell S., Lawrence A.J., Hoyer D., Jacobson L.H., Timmermann C., Castle D., Rossell S.L., Downey L.A., Pagni B.A., Galvão-Coelho N.L., Nutt D., Sarris J. (2023). Default mode network modulation by psychedelics: a systematic review. Int. J. Neuropsychopharmacol..

[167] Grinevich V., Knobloch-Bollmann H.S., Eliava M., Busnelli M., Chini B. (2016). Assembling the puzzle: pathways of oxytocin signaling in the brain. Biol. Psychiatry.

[168] Vohryzek J., Cabral J., Lord L.-D., Fernandes H.M., Roseman L., Nutt D.J., Carhart-Harris R.L., Deco G., Kringelbach M.L. (2024). Brain dynamics predictive of response to psilocybin for treatment-resistant depression. Brain Commun..

[169] Soares C., Gonzalo G., Castelhano J., Castelo-Branco M. (2023). The relationship between the default mode network and the theory of mind network as revealed by psychedelics – a meta-analysis. Neurosci. Biobehav. Rev..

[170] Smallwood J., Bernhardt B.C., Leech R., Bzdok D., Jefferies E., Margulies D.S. (2021). The default mode network in cognition: a topographical perspective. Nat. Rev. Neurosci..

[171] Avram M., Fortea L., Wollner L., Coenen R., Korda A., Rogg H., Holze F., Vizeli P., Ley L., Radua J., Müller F., Liechti M.E., Borgwardt S. (2024). Large-scale brain connectivity changes following the administration of lysergic acid diethylamide, d-amphetamine, and 3,4-methylenedioxyamphetamine. Mol. Psychiatr..

[172] Holze F., Singh N., Liechti M.E., D'Souza D.C. (2024). Serotonergic psychedelics—a comparative review comparing the efficacy, safety, pharmacokinetics and binding profile of serotonergic psychedelics. Biol. Psychiatry Cogn. Neurosci. Neuroimaging.

[173] Onofrj M., Russo M., Delli Pizzi S., De Gregorio D., Inserra A., Gobbi G., Sensi S.L. (2023). The central role of the Thalamus in psychosis, lessons from neurodegenerative diseases and psychedelics. Transl. Psychiatry.

[174] Griffiths R.R., Johnson M.W., Richards W.A., Richards B.D., McCann U., Jesse R. (2011). Psilocybin occasioned mystical-type experiences: immediate and persisting dose-related effects. Psychopharmacology.

[175] Geyer M.A. (2024). A brief historical overview of psychedelic research. Biol. Psychiatry Cogn. Neurosci. Neuroimaging.

[176] Singleton S.P., Wang J.B., Mithoefer M., Hanlon C., George M.S., Mithoefer A., Mithoefer O., Coker A.R., Yazar-Klosinski B., Emerson A., Doblin R., Kuceyeski A. (2022). Altered brain activity and functional connectivity after MDMA-assisted therapy for post-traumatic stress disorder. Front. Psychiatr..

[177] Sassenberg T.A., Safron A., DeYoung C.G. (2024). Stable individual differences from dynamic patterns of function: brain network flexibility predicts openness/intellect, intelligence, and psychoticism. Cerebr. Cortex.

[178] De Gregorio D., Aguilar-Valles A., Preller K.H., Heifets B.D., Hibicke M., Mitchell J., Gobbi G. (2021). Hallucinogens in mental health: preclinical and clinical studies on LSD, psilocybin, MDMA, and ketamine. J. Neurosci.: Offic. J. Soc. Neurosci..

[179] Timmermann C., Roseman L., Williams L., Erritzoe D., Martial C., Cassol H., Laureys S., Nutt D., Carhart-Harris R. (2018). DMT models the near-death experience. Front. Psychol..

[180] Timmermann C., Zeifman R.J., Erritzoe D., Nutt D.J., Carhart-Harris R.L. (2024). Effects of DMT on mental health outcomes in healthy volunteers. Sci. Rep..

[181] Silverstein S.M., Lai A. (2021). The phenomenology and neurobiology of visual distortions and hallucinations in schizophrenia: an update. Front. Psychiatr..

[182] Simonsson O., Carlbring P., Carhart-Harris R., Davis A.K., Nutt D.J., Griffiths R.R., Erritzoe D., Goldberg S.B. (2023). Assessing the risk of symptom worsening in psilocybin-assisted therapy for depression: a systematic review and individual participant data meta-analysis. Psychiatry Res..

[183] Gobbi G. (2024). CCNP Innovations in Neuropsychopharmacology Award: the psychopharmacology of psychedelics: where the brain meets spirituality. J. Psychiatry Neurosci.: J. Psychiatr. Neurosci..

[184] Vargas-Perez H., Grieder T.E., van der Kooy D. (2023). Neural plasticity in the ventral tegmental area, aversive motivation during drug withdrawal and hallucinogenic therapy. J. Psychoact. Drugs.

[185] Zhang M., Wang Y., Gao T.-M., Wang X. (2024). Psychedelics and consciousness: expanding the horizons of mind and therapy. Research.

[186] Rubin L.H., Carter C.S., Bishop J.R., Pournajafi-Nazarloo H., Drogos L.L., Hill S.K., Ruocco A.C., Keedy S.K., Reilly J.L., Keshavan M.S., Pearlson G.D., Tamminga C.A., Gershon E.S., Sweeney J.A. (2014). Reduced levels of vasopressin and reduced behavioral modulation of oxytocin in psychotic disorders. Schizophr. Bull..

[187] Rubin L.H., Carter C.S., Drogos L.L., Pournajafi-Nazarloo H., Sweeney J.A., Maki P.M. (2015). Effects of sex, menstrual cycle phase, and endogenous hormones on cognition in schizophrenia. Schizophr. Res..

[188] Goldman M., Marlow-O’Connor M., Torres I., Carter C.S. (2008). Diminished plasma oxytocin in schizophrenic patients with neuroendocrine dysfunction and emotional deficits. Schizophr. Res..

[189] de Vos C.M.H., Mason N.L., Kuypers K.P.C. (2021). Psychedelics and neuroplasticity: a systematic review unraveling the biological underpinnings of psychedelics. Front. Psychiatr..

[190] Heifets B.D., Olson D.E. (2024). Therapeutic mechanisms of psychedelics and entactogens. Neuropsychopharmacology.

[191] Werle I., Bertoglio L.J. (2024). Psychedelics: a review of their effects on recalled aversive memories and fear/anxiety expression in rodents. Neurosci. Biobehav. Rev..

[192] Cho M.M., DeVries A.C., Williams J.R., Carter C.S. (1999). The effects of oxytocin and vasopressin on partner preferences in male and female prairie voles (Microtus ochrogaster). Behav. Neurosci..

[193] Carter C.S. (2022). Sex, love and oxytocin: two metaphors and a molecule. Neurosci. Biobehav. Rev..

[194] Reed F., Foldi C.J. (2024). Do the therapeutic effects of psilocybin involve actions in the gut?. Trends Pharmacol. Sci..

[195] Stocker K., Liechti M.E. (2024). Methylenedioxymethamphetamine is a connectogen with empathogenic, entactogenic, and still further connective properties: it is time to reconcile “the great entactogen-empathogen debate.”. J. Psychopharmacol..

[196] Kirkpatrick M.G., Francis S.M., Lee R., de Wit H., Jacob S. (2014). Plasma oxytocin concentrations following MDMA or intranasal oxytocin in humans. Psychoneuroendocrinology.

[197] Bedi G., Hyman D., de Wit H. (2010). Is ecstasy an “empathogen”? Effects of ±3,4-methylenedioxymethamphetamine on prosocial feelings and identification of emotional states in others. Biol. Psychiatry.

[198] Ramos L., Hicks C., Caminer A., Couto K., Narlawar R., Kassiou M., McGregor I.S. (2016). MDMA ('Ecstasy’), oxytocin and vasopressin modulate social preference in rats: a role for handling and oxytocin receptors. Pharmacol. Biochem. Behav..

[199] Rowe J.W., Shelton R.L., Helderman J.H., Vestal R.E., Robertson G.L. (1979). Influence of the emetic reflex on vasopressin release in man. Kidney Int..

[200] Calabrese E.J., Dhawan G., Kapoor R., Iavicoli I., Calabrese V. (2015). What is hormesis and its relevance to healthy aging and longevity?. Biogerontology.

[201] Gems D., Partridge L. (2008). Stress-response hormesis and aging: “that which does not kill us makes us stronger.”. Cell Metab..

[202] Jong T. R. de, Menon R., Bludau A., Grund T., Biermeier V., Klampfl S.M., Jurek B., Bosch O.J., Hellhammer J., Neumann I.D. (2015). Salivary oxytocin concentrations in response to running, sexual self-stimulation, breastfeeding and the TSST: the Regensburg Oxytocin Challenge (ROC) study. Psychoneuroendocrinology.

[203] Nisbett K.E. (2024). Moxie begets MOXI:The journey to a novel hypothesis about Mu-opioid and OXytocin system Interactions. Comprehen. Psychoneuroendocrinol..

[204] Shadani S., Conn K., Andrews Z.B., Foldi C.J. (2024). Potential differences in psychedelic actions based on biological sex. Endocrinology.

[205] Strom J.O., Theodorsson A., Theodorsson E. (2011). Hormesis and female sex hormones. Pharmaceuticals.

[206] Ostrowski N.L., Lolait S.J. (1995). Oxytocin receptor gene expression in female rat kidney. The effect of estrogen. Adv. Exp. Med. Biol..

[207] Jiang J., Yang M., Tian M., Chen Z., Xiao L., Gong Y. (2023). Intertwined associations between oxytocin, immune system and major depressive disorder. Biomed. Pharmacother..

[208] Fahrbach S.E., Morrell J.I., Pfaff D.W. (1986). Effect of varying the duration of pre-test cage habituation on oxytocin induction of short-latency maternal behavior. Physiol. Behav..

[209] Molla H., Lee R., Tare I., de Wit H. (2023).

[210] Zeifman R.J., Wagner A.C., Monson C.M., Carhart-Harris R.L. (2023). How does psilocybin therapy work? An exploration of experiential avoidance as a putative mechanism of change. J. Affect. Disord..

[211] Higashida H., Hashii M., Tanaka Y., Matsukawa S., Higuchi Y., Gabata R., Tsubomoto M., Seishima N., Teramachi M., Kamijima T., Hattori T., Hori O., Tsuji C., Cherepanov S.M., Shabalova A.A., Gerasimenko M., Minami K., Yokoyama S., Munesue S.-I., Lopatina O. (2019). CD38, CD157, and RAGE as molecular determinants for social behavior. Cells.

[212] Peters S., Slattery D.A., Uschold-Schmidt N., Reber S.O., Neumann I.D. (2014). Dose-dependent effects of chronic central infusion of oxytocin on anxiety, oxytocin receptor binding and stress-related parameters in mice. Psychoneuroendocrinology.

[213] Quintana D.S., Guastella A.J. (2020). An allostatic theory of oxytocin. Trends Cognit. Sci..

[214] Lewis C.R., Tafur J., Spencer S., Green J.M., Harrison C., Kelmendi B., Rabin D.M., Yehuda R., Yazar-Klosinski B., Cahn B.R. (2023). Pilot study suggests DNA methylation of the glucocorticoid receptor gene (NR3C1) is associated with MDMA-assisted therapy treatment response for severe PTSD. Front. Psychiatr..

[215] Page C.E., Epperson C.N., Novick A.M., Duffy K.A., Thompson S.M. (2024). Beyond the serotonin deficit hypothesis: communicating a neuroplasticity framework of major depressive disorder. Mol. Psychiatr..

[216] Skosnik P.D., Sloshower J., Safi-Aghdam H., Pathania S., Syed S., Pittman B., D'Souza D.C. (2023). Sub-acute effects of psilocybin on EEG correlates of neural plasticity in major depression: relationship to symptoms. J. Psychopharmacol..

[217] Singewald N., Sartori S.B., Reif A., Holmes A. (2023). Alleviating anxiety and taming trauma: novel pharmacotherapeutics for anxiety disorders and posttraumatic stress disorder. Neuropharmacology.

[218] Li J.-R., Chiang K.-T., Kao Y.-C., Yu C.-L., Yang F.-C., Liang C.-S., Hsu T.-W. (2025). The association between study design and antidepressant effects in psychedelic-assisted therapy: a meta-analysis. J. Affect. Disord..

[219] Calabrese E.J., Mattson M.P. (2024). The catabolic—anabolic cycling hormesis model of health and resilience. Ageing Res. Rev..

